# Geological Substrate Effects on *Teucrium montanum* L. (Lamiaceae) Morphological Traits: Geometric Morphometrics Approach

**DOI:** 10.3390/plants12122381

**Published:** 2023-06-19

**Authors:** Nenad Zlatić, Sanja Budečević, Milan Stanković

**Affiliations:** 1 Department of Biology and Ecology, Faculty of Science, University of Kragujevac, Radoja Domanovića No. 12, 34000 Kragujevac, Serbia; 2 Department of Evolutionary Biology, Institute for Biological Research “Siniša Stanković”—National Institute of the Republic of Serbia, University of Belgrade, Blvd. depota Stefena 142, 11060 Belgrade, Serbia

**Keywords:** mountain germander, geometric morphometrics, morphological variability, substrate influence

## Abstract

The shape–environment relationship in plants refers to the ways in which the physical characteristics and structures of plants are influenced by their environment. Plants have evolved a remarkable ability to adapt to their specific habitats, and their shape and form play a crucial role in determining their survival and reproductive success. This study aimed to examine differences in size and shape between morphological traits in mountain germander (*Teucrium montanum* L.) from different geological substrates (calcareous and serpentinite). For this study, 400 individuals of *T. montanum* from 20 populations (ten populations from the serpentinite and ten from the calcareous substrate) were selected. Using the geometric morphometrics approach, it was shown that the degree of phenotypic variation in the size and shape of the corolla, leaf, and stem of *T. montanum* depends on the type of substrate. The main differences between the populations are the narrower part of the lower lip of the corolla, the narrower leaf, and the wider central part of the vascular system stem from serpentinite populations. The results of this study will contribute to a better understanding of the morphological variability of *T. montanum* in relation to edaphic conditions. In addition, the results confirm that certain morphological differences play an important role in the adaptive response in relation to substrate composition, especially for substrates with increased metal content, such as serpentinite. The shape–environment relationship in plants could define diversity and complexity in plant life, and underscores the importance of shape as a key factor in their survival and success in different habitats.

## 1. Introduction

Plant phenotypic variation refers to the ability of a plant to change its phenotype in response to environmental factors, including the type of substrate in which it grows [1,2,3,4]. Variation in the phenotypic expression of traits of a given organism under the influence of environmental factors affects its development and ecology [5,6,7]. This includes a variety of biotic and abiotic factors, which can influence phenotypic changes that are important for survival and reproduction in heterogeneous environments [1,2,8].

Stability in development allows the organism to resist developmental perturbations that may affect the variation in morphological structures. Variations in the size and shape of the different sides of bilaterally symmetrical structures, caused by developmental noise, result in fluctuating asymmetry (FA), the difference between the left and right sides of a bilaterally symmetrical organism [9,10]. Fluctuating asymmetry occurs mainly when developmental mechanisms are unable to control ontogenetic processes due to stress caused by certain environmental conditions. Therefore, fluctuating asymmetry is mainly used as an indicator of stress [11,12].

The allometric growth of plants represents a disproportionate growth that can change its shape during ontogenesis. In contrast, changes in shape that are not accompanied by changes in the size of morphological structures are referred to as non-allometric components. By separating allometric and non-allometric components, it is possible to identify changes in the shape of morphological components, independent of changes in size [13,14]. Allometric relationships are based on the observation that growth rates of different plant parts are not proportional to their size, but rather follow specific patterns that depend on various factors, such as genetic makeup, environmental conditions, and resource availability [15]. Allometric relationships are usually expressed in terms of scaling laws that describe the relationship between the size of one plant part and another, such as the size of leaves relative to the stem diameter [16]. These scaling laws can be used to predict how a plant’s growth and development will change in response to different environmental conditions or resource availability, and to compare the growth patterns of different plant species or individuals [15,17]. A common example of allometry in plants is the relationship between leaf size and stem diameter [16]. As a plant grows taller and larger, its stem diameter usually increases faster than its leaf size, resulting in a lower ratio between leaf and stem size. This is because the stem supports and transports the growing plant, while the leaves are primarily responsible for photosynthesis and energy production [18,19].

Edaphic factors determine the occurrence of various morpho-anatomical and ecophysiological traits [20]. Due to the evolution of complex mechanisms to adapt to a particular substrate type, the distribution of some ecological groups is completely determined by the substrate type [21]. Some groups are characterized by a certain degree of tolerance to different edaphic conditions, with obligate and facultative ecological groups, i.e., edaphic ecotypes, being distinguished according to the substrate type [22]. 

Soil is a type of substrate that plays a crucial role in the growth and development of plants. The properties of the soil can have a significant impact on the morphology, or physical form and structure, of plants. The availability of nutrients in the substrate can influence plant growth and morphology. For example, plants growing in nutrient-poor substrates may exhibit stunted growth and smaller leaves, while plants growing in nutrient-rich substrates may exhibit more vigorous growth and larger leaves [23]. The amount of water available in the substrate can also affect plant growth and morphology. Plants grown in dry substrates may exhibit smaller leaves and a more compact growth habit, while plants grown in moist substrates may exhibit larger leaves and a more spreading growth habit [24,25]. The substrate pH can also affect plant growth and morphology. If the pH of the substrate is not suitable for a particular plant species, the plant may exhibit stunted growth and other abnormalities [26]. The mineral composition of the substrate can also affect plant growth and morphology. For example, plants grown in substrates high in calcium may have stronger stems and thicker leaves, while plants grown in substrates high in potassium may bear larger fruits or flowers [23,27].

Serpentinite soils are characterized by high levels of heavy metals, low levels of essential nutrients, and high pH, which can create challenging conditions for plant growth [28]. As a result, many plant species that grow in serpentinite or ultramafic soils have evolved specific morphological and physiological characteristics, to tolerate and even thrive in these harsh environments [21]. Plants growing in serpentinite soils often have smaller leaves than plants growing in other soil types. This may be due to the fact that the high concentrations of heavy metals in the soil can affect the leaf development and growth. To reduce water loss, and protect against heavy-metal toxicity, plants growing in serpentinite soils often have thicker leaf cuticles than plants growing in other soil types. Plants growing in serpentinite soils often have extensive root systems that allow them to explore a larger soil volume in search of nutrients and water. In some cases, plants growing in serpentinite soils may exhibit a dwarf phenotype, with shorter stems and smaller overall size. This may be due to low nutrient availability, and other stresses associated with these soils [28,29,30,31,32,33,34].

Calcareous soils are characterized by high levels of calcium carbonate and a high pH, which can be particularly challenging for plant growth [27]. However, many plant species have evolved modifications to thrive in these conditions. Plants growing in calcareous soils often have thicker leaves than plants growing in other soil types. This is because the high pH and calcium carbonate content of the soil can cause water stress in plants, which can be alleviated by thicker leaves [35]. The high pH of calcareous soils can also limit the availability of some important nutrients, such as iron and manganese, making it difficult for plants to extract these nutrients from the soil [36]. As a result, some plants growing in calcareous soils may have smaller root systems than those growing in other soil types. Some plants growing in calcareous soils form cluster roots, which are dense, branched structures that can secrete organic acids to dissolve calcium carbonate and release essential nutrients [37]. In some cases, plants growing in calcareous soils may produce more seeds than plants growing in other soil types. This may be because the high pH and calcium content of the soil can enhance seed production and germination [38].

Since the species *Teucrium montanum* is a facultative serpentinophyte that can also grow on calcareous geological substrate, it is necessary to intensify the ecological differentiation of this species, in terms of the type of geological substrate. Due to its specific distribution depending on substrate type, *T. montanum* has been a relevant species for comparative analysis of the effects of the substrate on the content of elements [22] and radionuclides in soil and plant material [39], as well as variability in the content of essential oil components [40] and their potential role in adaptation responses. The results of these studies suggest that there is variability in ecophysiological traits in relation to the habitat substrate, and that their potential importance in adaptation responses is not yet fully understood, as well as the phylogenetic relationship to its closest relatives [41].

Here, we tested the hypothesis that the substrate influences the ecological differentiation and the changes in the shape and size of the corolla, leaf, and stem of *T. montanum* in relation to the geological substrate. The main objective of the study is to determine the differences in the size and shape of the corolla, leaf, and cross-section of the stem of *T. montanum* populations developed on different geological substrates (calcareous and serpentinite). Overall, the influence of serpentinite soils on plant morphology is a good example of how plants adjust to extreme environments. Differences in the shape of the corolla, leaves, and stem of populations sampled from serpentinite substrate are expected to primarily be differences in the size of morphological traits due to the specific chemical composition of the serpentinite substrate, which is reflected in a deficiency of macronutrients and the presence of large amounts of heavy metals. By studying how plants have evolved to cope with these challenging conditions, we can gain a better understanding of plant biology, and ecology more broadly.

## 2. Materials and Methods

### 2.1. Plant Material

**Figure 1:**
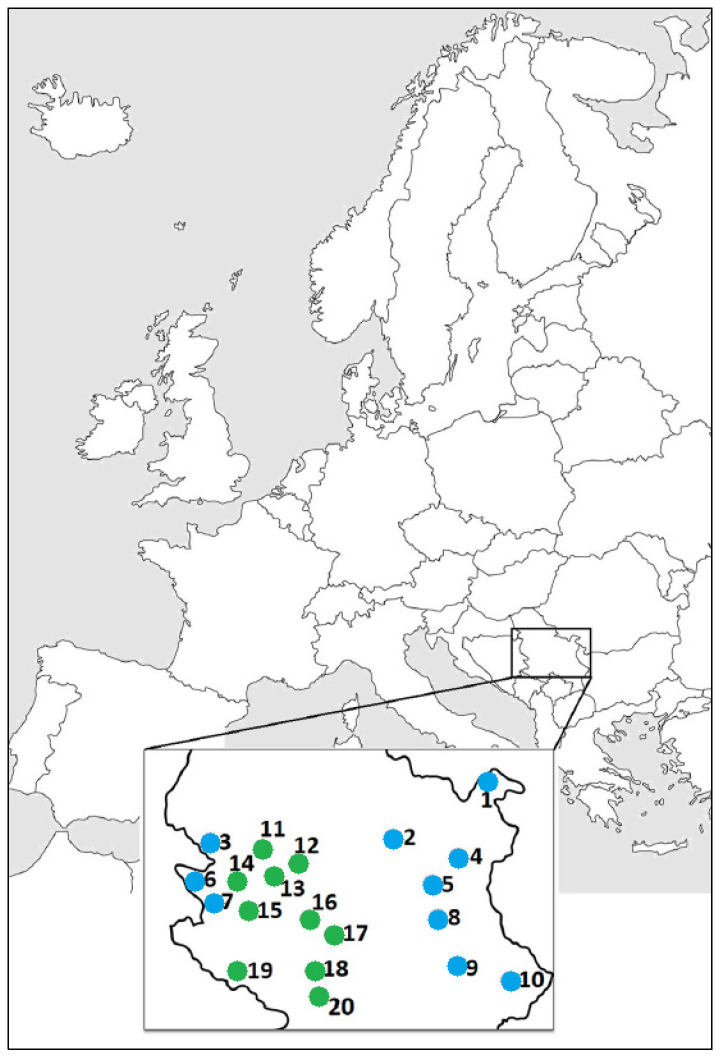
The distribution of the *T. montanum* sampling localities within the territory of Serbia. The numbers correspond to the list of localities in Table 1. (Calcareous localities 1–10/blue; Serpentinite localities 11–20/green).

For this study, 400 individuals of *T. montanum* from 20 populations (ten populations from the serpentinite and ten from the calcareous substrate) were selected. Twenty individuals were sampled from each population, in the flowering phase during 2016 and 2017 (Figure 1; Table 1). To minimize the clone selection risk, all sampled individuals from selected populations were located at least 10 m from each other. To reduce inter-individual variability, only plants of a similar size were selected. The samples were identified and deposited at the University of Kragujevac (Serbia), Faculty of Science, Department of Biology and Ecology.

### 2.2. Preparation of Plant Structures for Geometric-Morphometric Analyzes

**Figure 2:**
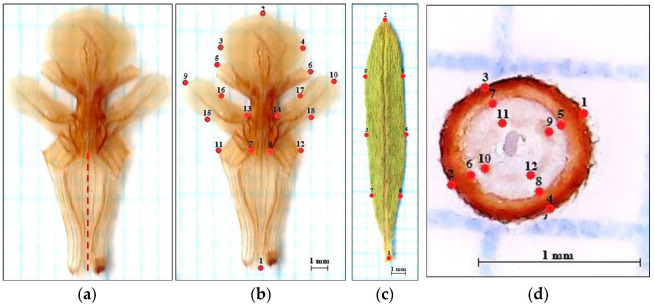
The preparation of plant structures for analysis by geometric morphometrics. (**a**) The dissection of the flower tube. (**b**) The position of specific points on the corolla, as follows: (1) the end of the central nerve, (2) the top of the central nerve, (3) the widest part of the left side of the lower lip, (4) the widest part of the right side of the lower lip, (5) the end of the left peripheral nerve of the middle lip, (6) the end of the right peripheral nerve of the middle lip, (7) the section of the left peripheral nerve and the end of the left upper lip, (8) the section of the right peripheral nerve, (9) the end of the left peripheral nerve, (10) the end of the right peripheral nerve, (11) the left margin at point 7, (12) the right margin at point 8, (13) the junction between the left upper lip and left middle lip, (14) the junction between the right upper lip lips and right middle lip, (15) the narrowest part of the left upper lip, (16) the narrowest part of the left upper lip at point 15, (17) the narrowest part of the upper right lip at point 18, (18) the narrowest part of the right upper lip. (**c**) The leaf: (1) the end of the central nerve, (2) the tip of the central nerve, (3) the left half of the length between points 1 and 2, (4) the right half of the length between points 1 and 2, (5) the left half of the length between points 2 and 3, (6) the right half of the length between points 2 and 4, (7) the left half of the length between points 1 and 3, (8) the right half of the length between points 1 and 4. (**d**) The cross section of the stem: (1) the widest part of the right side of the cross section, (2) the widest part of the left side of the cross section, (3) the highest part of the left and right half of the cross section, (4) the lowest part of the left and right side of the cross section, (5) the widest part of the right side of the phloem at the level of point 1, (6) the widest part of the left side of the phloem at the level of point 2, (7) the highest part of the left and right half of the phloem at the level of point 3, (8) the lowest part of the left and right half of the phloem at the level of point 4, (9) the widest part of the right side of the xylem at the level of point 5, (10) the widest part of the left side of the xylem at the level of point 6, (11) the highest part of the left half and the right side of the xylem at the level of point 7, (12) the sides of the xylem at the level of point 8.

The 800 flowers were used to determine the pattern of variation in the size and shape of the corolla of *T. montanum*. Freshly sampled flowers were placed in labeled plastic jars, 50 mL in volume, which were filled with 70% ethanol. The plant material was stored in a box at a temperature of 20 °C until dissection. The separation of flower structures was performed by one incision through the flower tube, at the junction of the upper two lips (Figure 2a). After dissection, the flower parts were placed on a glass plate, onto which 50% glycerin had been previously added. The flower parts were stretched with tweezers to their natural shape. After this procedure, parts of the flower were scanned with a scanner (Epson Perfection V19) at a resolution of 600 dpi. To determine the size and shape, 18 landmarks were defined (Figure 2b). The placement of specific landmarks on the flower corolla was performed using the software program tpsDig2 [42].

The 1200 leaves were used to determine the pattern of variation in the size and shape. Freshly sampled leaves were placed into labeled cellulose bags, in which they were dried and pressed until being scanned. Leaves in the middle zone of the shoot were used for scanning. Three leaves were randomly selected from three shoots from one individual, from the central part of the habitus. The dehydrated leaves were scanned at a resolution of 600 dpi. To determine the size and shape of the leaves, 8 landmarks were defined (Figure 2c). 

To determine the variation in the size and shape of the stem, 1200 cross-sections of the stem were used. Stem dissection was performed using a razor blade. Cross-sections of the stem were made in the area of the middle part of the stem. After this procedure, cross-sections of the stem were photographed under a light microscope (Nikon Ti-Eclipse), at a resolution of 96 dpi. To determine the shape and size of the stem, 12 landmarks were defined (Figure 2d). 

### 2.3. Statistical Analyses

Procrustes superimposition was used to extract shape information, and eliminate effects of rotation, translation, and position. The centroid size (CS) was used as a measure of the size of *T. montanum* plant structures. To detect the variability of the symmetric and asymmetric components of variation in the shape of *T. montanum* plant structures, we used principal component analysis (PCA). Mathematically, the input data for the calculation of principal components in geometric morphometry are the covariance matrices of the coordinates of certain points. The significant difference in the shape of plant structures from different geological substrates was tested using discriminant function analysis (DFA). The difference between the mean shapes of the different plant structures of *T. montanum* was tested using the total variation in shape. To eliminate differences between the mean shapes caused by size changes, DFA was repeated, using the residual values of the multivariate regression of the shape variables on the centroid size as input data. The statistical significance of differences between the mean shapes of pairs of specific plant structures was determined by permutation tests with 10,000 iterations, using the Procrustes distances as input data. To determine the presence of allometry, i.e., the relationship between the size and shape of plant structures, a multivariate regression of the shape variables on the log value of the size of the centroid (log CS) was performed. The obtained percentage of the total variation in plant structures that can be explained by the change in their size describes the dependence of the variation in the shape of plant structures on their size. Statistical significance against the null hypothesis of independence of shape and size was tested by a permutation test with 10,000 iterations. All analyses were performed using the MorphoJ software package [43]. 

## 3. Results

### 3.1. The Influence of the Geological Substrate on the Variation in Size and Shape in the Corolla, Leaf, and Stem of T. montanum 

The mean values of the centroid size (CS) for the examined plant structures are presented in Table S1. Using a one-factor analysis of variance, it was shown that there was a significant statistical difference between the average centroid size for the leaf and the stem, while no statistically significant difference was observed for the centroid size of the corolla (Table S2). Applying the Tukey post hoc test, it was shown that there were statistically significant differences in the size of the leaf and stem from calcareous and serpentinite, while there was no statistically significant difference in the size of the corolla between these two types of habitat (Table S3).

### 3.2. Variation in the Shape of the Plant Structures of T. montanum

The variation in the shape of the reproductive and vegetative plant structures in the populations of the *T. montanum* from habitats with calcareous and serpentinite geological substrate was evaluated using principal component analysis (PCA). Based on the obtained results, it was shown that the highest percentage of shape variation could be described by the first three PC axes for the analyzed plant structures.

### 3.3. Symmetric and Asymmetric Components of Corolla Shape Variation

**Figure A1:**
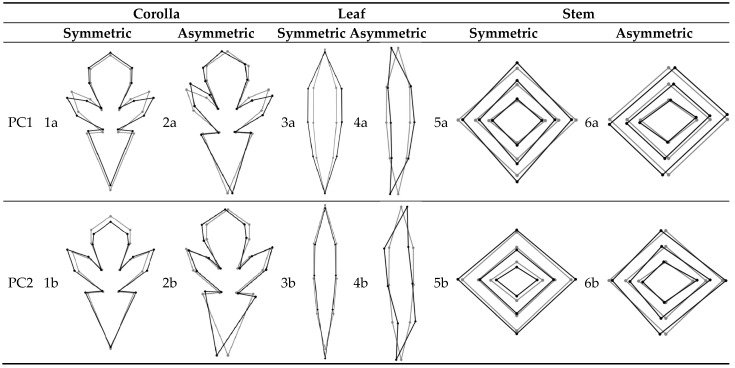

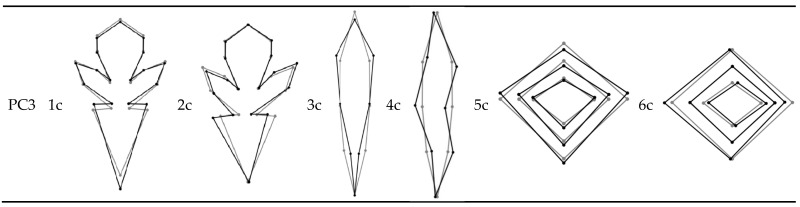
Changes in the pattern of variation in the shape of the corolla, leaf, and stem of *T. montanum* in symmetric and asymmetric components (**PC1**–**PC3**). A shape change in a positive direction is shown by a solid black line with filled circles, while a change in a negative direction is shown by a solid gray line with filled circles.

The variation in the symmetric component of the first PC axis is described by 25.3% of the corolla shape variation (Table S4). The variation in the shape described by the first PC axis refers to the expansion of the middle and lateral lobes of the lower lip, and the elongation of the upper lobes of the upper lip, in the positive part of the PC axis (Figure A1 and Figure 1a). The second PC axis describes 19.4% of the corolla shape variation. The variation in the shape of the corolla is described by the second PC axis, referring to the narrowing of the middle and lateral lobes of the lower lip in the positive direction, and the widening and lengthening of the upper lobes of the upper lip in the positive direction, of the PC axis (Figure A1 and Figure 1b). The third PC axis describes 13.2% of corolla shape variation. The variation in the shape of the corolla described by the third PC axis refers to the shortening of the middle lobe of the lower lip, the narrowing of the lateral parts of the upper lobes of the upper lip, and the expansion of the base of the corolla, in the positive direction of the PC axis (Figure A1 and Figure 1c).

The variation in the asymmetric component of the first PC axis is described by 31.6% of the corolla shape variation (Table S4). The variation in the shape is described by the first PC axis, which refers to a slight tilting of the middle and lateral lobes of the lower lip to the left, i.e., a tilting of the upper lobes of the upper lip and the base of the corolla to the right, in the positive direction of the PC axis (Figure A1 and Figure 2a). The shape variation of the second PC axis was described by 24.8% of the shape variation. The variations refer to the slight bending of the middle, lateral lobes of the lower lip and the base of the corolla to the left side, as well as the lengthening of the right side of the upper lobes of the upper lip, and the shortening of the left side of the upper lobes of the upper lip, in the positive direction of the PC axis (Figure A1 and Figure 2b). The shape variation of the third PC axis is described by 7.9% shape variation. The variation refers to the tilting of the base of the corolla to the left side, as well as the lengthening and narrowing of the right side of the upper lobes of the upper lip, and the shortening and widening of the left side of the upper lobes of the upper lip, in the positive direction of the PC axis (Figure A1 and Figure 2c).

### 3.4. Symmetric and Asymmetric Components of Leaf Shape Variation

**Figure 3:**
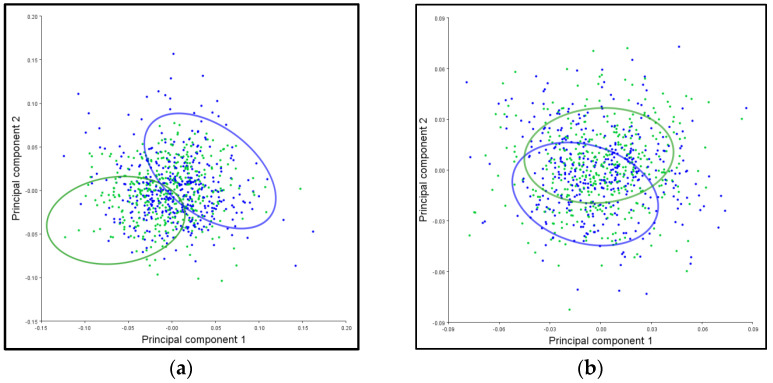
Influence of the substrate on the differentiation of the *T. montanum* corolla shape from calcareous (blue ellipse) and serpentinite (green ellipse) habitats. The graph represents 95% confidence ellipses for the average values of the (**a**) symmetric and (**b**) asymmetric components of corolla shape variation.

The variations in the symmetric component of the first PC axis are described by 65.5% of the variation in leaf shape (Table S5). The variations in the leaf shape described by the first PC axis refer to the expansion of the upper and lower halves, as well as the shortening of the upper half, in the positive direction (Figure A1 and Figure 3a). The second PC axis describes 13.2% of leaf shape variations. The variations in the shape of the leaf described by the second PC axis refer to the narrowing of the upper and lower half of the leaf, as well as to the elongation of the lower half, and shortening of the upper half of the leaf, in the positive direction (Figure A1 and Figure 3b). The third PC axis describes 9.6% of leaf shape variations. The variations in the shape of the leaf described by the third PC axis refer to the expansion and shortening of the upper half, and to the narrowing of the lower half of the leaf, in the positive direction of the PC axis (Figure A1 and Figure 3c).

**Figure 4:**
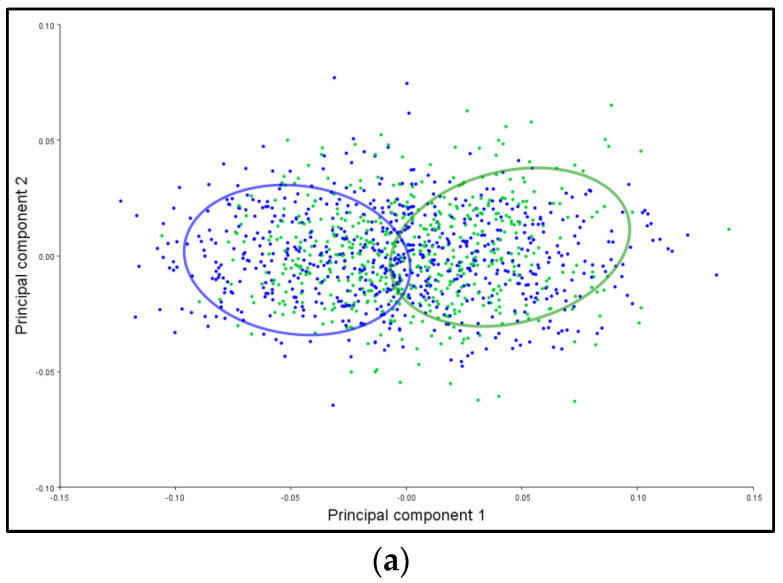

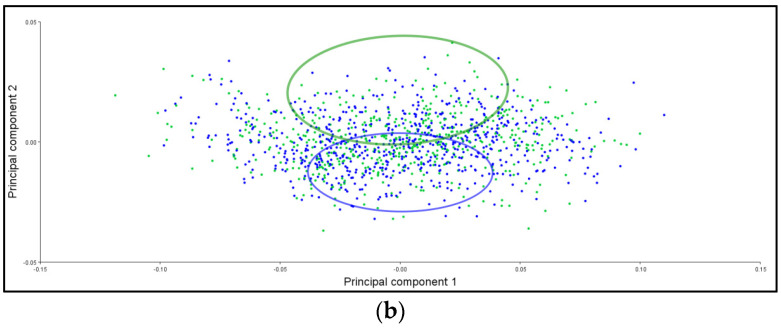
The influence of the substrate on the differentiation of *T. montanum* leaf shape from calcareous (blue ellipse) and serpentinite (green ellipse) habitats. Confidence ellipses of 95% are shown for the average values of the (**a**) symmetric and (**b**) asymmetric components of leaf shape variation.

The variation in the asymmetric component of the first PC axis is described by 85.9% of the variation in leaf shape (Table S5). The variation in the shape of the leaf described by the first PC axis refers to the tilting of the upper and lower half of the leaf to the left, and the central part to the right, in the positive direction of the PC axis (Figure A1 and Figure 4a). The shape variation of the second PC axis is described by 7.9% shape variation. The variation refers to the tilting of the upper half to the right and the lower half to the left, as well as the tilting of the middle half of the upper part to the left, and the middle half of the lower part to the right, in the positive direction of the PC axis (Figure A1 and Figure 4b). The shape variation of the third PC axis is described by 2.8% shape variation. The variations refer to the tilting of the upper, middle, and lower parts to the left side, as well as the tilting of the middle half of the lower and upper parts to the right side, in the positive direction of the PC axis (Figure A1 and Figure 4c).

### 3.5. Symmetric and Asymmetric Components of Stem Shape Variation

**Figure 5:**
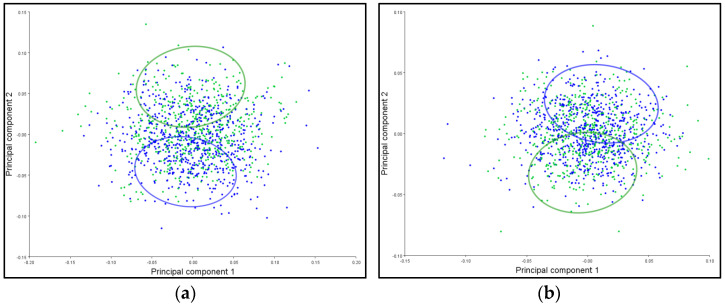
The influence of the substrate on the differentiation of *T. montanum* stem from calcareous (blue ellipse) and serpentinite (green ellipse) habitats. Shown are 95% confidence ellipses for the average values of the (**a**) symmetric and (**b**) asymmetric components of stem shape variation.

The variation in the symmetric component of the first PC axis is described by 42.8% of the variation in stem shape (Table S6). The variation in the shape of the stem described by the first PC axis refers to the elongation of the upper and lower half of the cuticular, phloem, and xylem elements, as well as the narrowing of the central part of the mentioned elements, in the positive direction of the PC axis (Figure A1 and Figure 5a). The second PC axis describes 26.2% of the variation in stem shape. The variations in the shape of the stem described by the second PC axis refer to the elongation of the cuticular elements in the lower, middle, and upper parts, as well as the narrowing of the phloem and xylem elements in the upper, lower, and middle parts, in the positive direction of the PC axis (Figure A1 and Figure 5b). The third PC axis describes 11.1% of the variation in stem shape. The variations in the shape of the stem described by the third PC axis refer to the narrowing of the upper half of the cuticular, phloem, and xylem elements, to the expansion of the lower half of the mentioned elements, and the tilting of all elements toward the upper half, in the positive direction of the PC axis (Figure A1 and Figure 5c).

**Figure 6:**
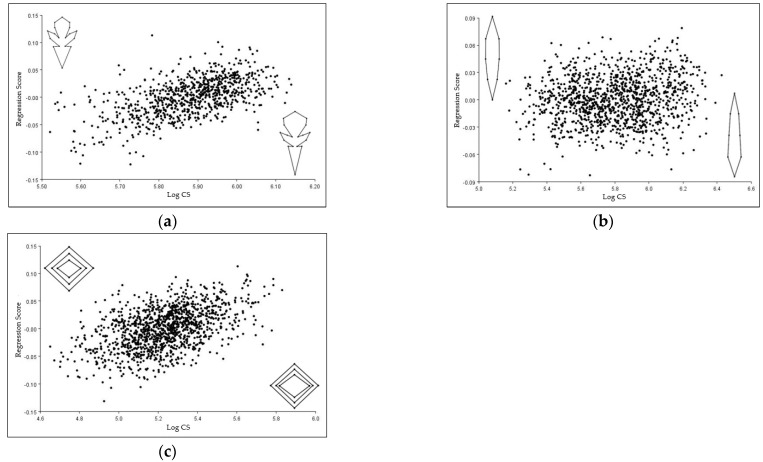
The multivariate regression of shape variables on centroid size (CS) in the (**a**) corolla, (**b**) leaf, and (**c**) stem of *T. montanum*. Scatter diagrams of regression scores are presented, in which there are shape models with the maximum value (lower right corner) and with the minimum CS value (upper left corner).

The variations in the asymmetric component of the first PC axis are described by 40.1% of the variation in stem shape (Table S6). The variations in the shape of the stem described by the first PC axis refer to the tilting of the upper half of the cuticular, phloem, and xylem elements to the right side, and the tilting of the lower half of the mentioned elements to the left side, as well as the movement of the right side of the central part up, and the left side up, in the positive direction PC axes (Figure A1 and Figure 6a). The shape variation of the second PC axis was described by 23.9% of the shape variation. The variations refer to the tilting of all examined elements of the upper and lower half to the left side, as well as the lengthening and narrowing of the central part to the right side in the positive direction of the PC axis (Figure A1 and Figure 6b). The shape variations of the third PC axis are described by 11.0% shape variation. The variations are related to the lengthening of the upper, middle, and lower part of the cuticular elements, and their narrowing to the left side, in the positive direction of the PC axis. In addition to the mentioned variation, a shift of all parts of the xylem element to the right side in the positive direction of the PC axis is observed (Figure A1 and Figure 6c).

### 3.6. PCA Ellipses

The patterns of corolla shape variation for the symmetric and asymmetric components are presented in Figure 3. The main characteristic of both shape variation components overlaps to a small extent for the symmetric component (Figure 3a), while for the asymmetric component, the overlap of the ellipses is much more pronounced (Figure 3b). Since the confidence ellipses include more than 95% of the mean values of the sample, this way of displaying the results indicates that there is a statistically significant difference between the shape of the corolla for the symmetric (*p* < 0.0001) and the asymmetric component (*p* < 0.0001).

The patterns of leaf shape variation for the symmetric and asymmetric components are shown in Figure 4. The confidence ellipses partially overlap for the symmetric component (Figure 4a), while the overlap is more pronounced for the asymmetric component (Figure 4b). The results presented in Figure 4 show that there is a statistically significant difference between the leaf shapes for the symmetric component (*p* < 0.0001) of the *T. montanum* species in relation to the type of habitat, while there is no significant difference for the asymmetric component (*p* < 0.5630).

The patterns of stem shape variation for the symmetric and asymmetric components are presented in Figure 5. The main characteristic of both shape variation components is that the confidence ellipses for the symmetric component are completely separated (Figure 5a), while for the asymmetric component, they slightly overlap (Figure 5b). Based on the results presented in Figure 5, it was observed that there is a statistically significant difference between the shape of the stem for the symmetric component (*p* < 0.0001), while for the asymmetric component, there is no significant difference (*p* < 0.1200) concerning the type of habitat.

### 3.7. Allometric Shape Differences in the Corolla, Leaf, and Stem of T. montanum

A statistically significant influence of allometry was determined for the symmetric component of all three analyzed plant structures (*p* < 0.01). Allometry accounted for 5.83% of corolla shape change, and 6.14% of stem shape change. Although significant, only 0.38% described the dependence of shape on leaf size, representing a small proportion of the total morphological variation (Table S7).

The influence of allometry on the variation in the shape of the corolla could be seen as the widening of the middle lobe, and the narrowing of the base of the corolla (Figure 6a). In the case of the leaf, with the increase in size, the upper half of the leaf narrowed, and the lower half of the leaf expanded (Figure 6b). In the cross-section of the stem, with increasing size, the xylem element expanded in all directions (Figure 6c).

### 3.8. Discriminant Analysis

The differences caused by variation in the shape of the corolla, leaf, and stem of *T. montanum* collected from calcareous and serpentinite substrates were determined by using discriminant analysis (DFA), with and without an allometric component (Table S8). Statistically significant differences were observed for all tested plant structures, with allometry and without allometry, for the symmetric components of corolla, leaf, and stem. Based on the obtained results, it was shown that allometry showed no significant effect on the differences in the shape of the examined plant structures.

**Figure 7:**
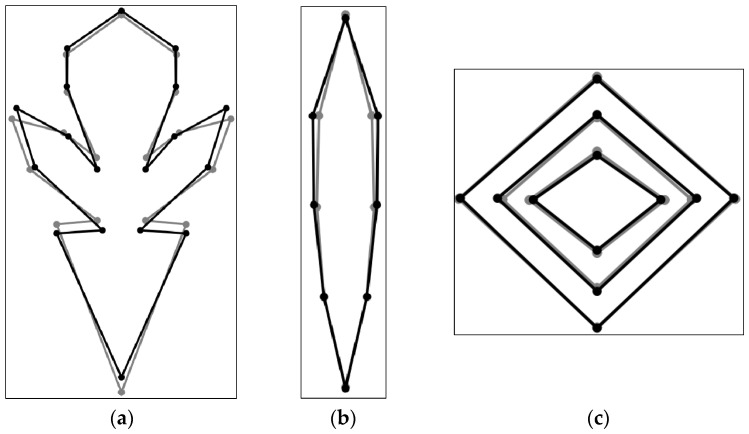
Diagrams of the examined plant structures with statistically significant differences in allometric components are presented. Diagrams of the (**a**) corolla, (**b**) leaf, and (**c**) stem are presented. The shape variation in calcareous populations is presented as a solid black line with filled circles, while the variation in serpentinite populations is presented as a solid gray line with filled circles.

By comparing the mean shapes of the examined plant structures obtained by discriminant analysis on the residuals, their differences were established, which are mainly manifested through the expansion and narrowing of structural elements (Figure 7). Variations in the mean shape of the corolla in the population from calcareous habitats are related to the slight expansion of the middle lobe of the lower lip, the lengthening of the upper lobes of the upper lip of the leaf, and the narrowing of the base of the corolla (Figure 7a). Differences in leaf shape relative to mean shape for calcareous populations relate to the spread of the upper half of the leaf, relative to serpentinite populations (Figure 7b). The mean shapes of stems from different geological substrates differ in width. The phloem elements of the stem from the calcareous substrate are slightly wider compared to those from the serpentinite one, while the xylem elements from the calcareous substrate are narrower than those from the serpentinite one (Figure 7c).

## 4. Discussion

The geological substrate and chemical profile of the soil have a deleterious effect on plant survival and growth, and heavy metal toxicity can have evolutionary and ecological effects [44]. Various environmental factors may alter the anatomical, morphological, and ecophysiological traits of plants from an evolutionary-ecology perspective [45]. It should be noted that the interaction of plants with the substrate indicates differences in species responses to different environments. Moreover, it is suggested that differentiation in the responses of plant species to changes in the environment, with variability in traits within a species, is not only significant, but also species-specific [3]. 

In general, plants could respond to certain environmental factors in the same way [3]. However, our results indicate that differences in the average shapes of corolla, leaf, and stem of *T. montanum* differ from each other. More precisely, literature data suggest that the genome of *T. montanum* from different habitats is plastic with respect to xeromorphic traits [43]. For example, *T. montanum* from calcareous and serpentinite habitats exhibits specific features in the morphological structure, while the same in plants from serpentinite are referred to as serpentinomorphs [46].

Symmetry is recognized as an essential feature of the morphological and anatomical organization of plant organisms [47,48,49,50], and it is involved in numerous aspects of morphological variations, and responses to abiotic and biotic stresses [49]. In addition, the presence of asymmetry in relation to the substrate represents the response of plants to a particular environmental factor. Symmetric or asymmetric components are generally associated with perturbations caused by environmental factors [49,51]. Fluctuating asymmetry is also caused by variations in response to the specific environment [52]. The plant parts are constantly exposed to environmental stresses during their development, and variability is the mechanism that could cause fluctuating asymmetry, as an outcome of developmental instability. In agreement with literature data, the manifestation of fluctuating asymmetry is the consequence of a developmental noise that usually occurs on one of the different sides of bilaterally symmetrical structures, i.e., in the case of *T. montanum*, single photosynthetic and conducting organs. Recent experimental data have confirmed the theoretical assumption that fluctuating asymmetry is not only the result of developmental instability, but phenotypic variation as the response of the developmental system to different environmental factors occurring during the developmental process [53]. Accordingly, the causes of fluctuating asymmetry are entirely non-genetic [53]. Patterns of asymmetry arising from responses to environmental heterogeneity would reflect the spatial distribution of heterogeneity in relevant microenvironmental factors [3,48]. It was found that the extent of fluctuating asymmetry of the studied biological structures was greater in the photosynthetic and conductive organs of the calcareous plants, in contrast to the serpentinite ones. Excess nutrients in plants can simultaneously increase growth and asymmetry. Namely, Martel et al. [54] found that leaves that grew faster had higher levels of asymmetry, which is consistent with our results. Comparing the size of fluctuating asymmetry in leaves and flowers in 19 European species, Møller and Ericsson [55] found that the size of fluctuating asymmetry was similar between the vegetative and reproductive organs of the analyzed plants. However, recent studies by Sandner and Matthies [56] showed that although stress affected the reduction of biomass and number of flowers in *Silene vulgaris*, the fluctuating asymmetry list of this species did not increase under stressful conditions. In this case, the leaf was not the most reliable source of environmental stress, unlike the flower, whose symmetry was under great selection pressure, so it could be a better measure of developmental instability in plants, while our results showed the opposite, which largely depended on the biology and ecology of the examined species. In this case, morphological variation could be used as an indicator of population genetic stability by acting as a protective factor [48]. For example, Tucić et al. [57] showed that phenotypic plasticity in *Iris pumila* contributes to morphological asymmetry, which is a response to the heterogeneity of environmental factors. The same conclusions were reached by other authors [48,58,59]. 

According to our results, it could be concluded that the changes in the mean shape of the corolla of the population from calcareous habitats are related to the slight enlargement of the middle lobe of the lower lip, the elongation of the upper lobes of the upper lip leaflets, and the narrowing of the base of the corolla. This supports the contention that the presence of certain macro- and micronutrients in the substrate significantly affects the flower size and shape [60]. The variations in flower morphology caused by differences in the soil chemical composition could have implications for plant reproduction [60]. Soil macronutrients such as Ca, Mg, N, P, and K influence flower size and number [60,61,62]. The presence of heavy metals in the soil often results in reduced flower growth [62] and number [63]. In addition, contaminated environments have different concentrations of pollutants which, when absorbed into tissues, affect plant growth. This can affect the size and shape of plant parts [47]. For example, Vujić et al. [47] found that the petals of *Iris pumila* were shorter and wider in the polluted environment than in the unpolluted environment. Some findings confirm that pollutants reduce plant growth, and have a negative effect on reproductive biology and flower morphology [64,65,66]. Moreover, Meindl et al. [67] showed that the serpentinite soil composition affects the flower size of *Mimulus guttatus*. Individuals from serpentinite soils had 60% shorter inflorescences, 12% fewer petals, and 52% fewer open flowers per inflorescence than individuals from non-serpentinite habitats [67]. Previous studies confirmed the direct effect of serpentinite soils on inflorescence size and height, in response to the lower amount of nutrients in the substrate [67]. The presence of heavy metals such as Ni in the substrate has a negative effect on plant reproduction [68]. In plants, the evolutionary response to environmental stress is slow growth, and consequently low nutrient requirements, with the drawback of lower overall productivity. This conservative strategy is referred to as “stress resistance syndrome”, and is common among plants in arid areas. This strategy, used by serpentinophytes, allows homeostasis to be maintained in their vegetative structures. This strategy allows the plant to perform sufficient photosynthesis to produce developmentally stable reproductive organs [69].

The results of this study show that the leaves of populations from serpentinite are smaller and narrower in contrast to populations from calcareous substrate. These differences are consistent with the studies mentioned earlier. For example, Dudić et al. [70] studied the morphological parameters of serpentinophytes, and found that obligate serpentinophytes had poorly developed branches, thin leaves, and a xeromorphic structure. The palisade tissue and cuticle were well developed, while the cell walls of the epidermal cells were strongly thickened [70]. The overlapping structural features represent the response of the plants to the specific conditions of arid habitats, which are characterized by intense insolation, high temperatures, and lack of water in the substrate. In obligate (*Fumana bonapartei*) and facultative (*Seseli rigidum*) serpentinophytes, a multilayered palisade tissue is formed, representing a response to the intense insolation in serpentinite habitats [70]. Leaves from serpentinite plants are small, covered with hairs, and slightly curved on the abaxial side. In the cross-section, the leaves are thicker compared to serpentinite populations, and have a distinct cuticle. The leaves of plants from serpentinite are smaller and broader than those of calcareous plants, while the leaf margins are strongly curved toward the abaxial side. In plants from serpentinite habitats, xeromorphic features are strongly expressed in association with the serpentinite substrate, and the effect of physical drought [46]. Moreover, Vieira et al. [71] showed that there are differences in leaf shape between populations of the same species. For example, Adamidis et al. [72] studied 17 plant species occurring in serpentinite and non-serpentinite habitats. The same authors indicated that the specific leaf area, leaf length, and leaf width had higher values in non-serpentinite habitats, while the percentage of dry matter, and the leaf thickness, had higher values in species from serpentinite. The leaf width and length are known to be lower in arid and nutrient-poor habitats, such as serpentinite [73]. Species from serpentinite habitats provide more structural compound resources, and therefore the leaves are thicker and have a smaller specific surface area of the leaf blade. Considering that leaf thickness is related to water retention [74], the higher values of this trait in plants from serpentinite habitats could be related to the low water retention capacity [75]. The author Veličković [76] compared leaves of the species *Tilia cordata*, from a contaminated site and another site without pollutants. The results showed that leaves were smaller in the contaminated area, indicating slow growth and plant development [76]. Moreover, Pollicelli et al. [77] investigated the effect of heavy metals in the soil on the leaf size and shape of *Cressa truxillensis* from salt marshes. They found that leaf size in the species studied was not affected by heavy-metal levels in the soil, but shape was. The sites with higher concentrations of elements such as Zn, Pb, and Cu resulted in lance-shaped leaves, while the site with the lowest concentration of soil metals resulted in spherical leaves [77]. It was found that the high concentrations of Ni and Pb in the leaves of *Iris pumila* in a polluted area were about six times higher than in an unpolluted area [78]. This could be the reason for the reduction in length and size in our studied leaves. Moreover, it was shown that variations in the symmetrical components of leaves differed in two species and their hybrids, depending on the effect of certain environmental factors [79]. Environmental factors were shown to have a greater influence on ecophysiological traits and leaf phenology than genetic variation [51,80]. 

According to the results of this study, the cross sections of plant stems from different geological substrates differed in size and width. The xylem elements of the stem from the calcareous substrate were narrower compared to those from the serpentinite substrate, while the phloem elements of the stem from the calcareous substrate were slightly wider compared to those of the stem from the serpentinite substrate. For example, Dudić et al. [70] pointed out that the species *Seseli rigidum* from serpentinite habitats was taller, and the stem was more developed, than the species from calcareous habitats. The same authors indicated that the reason is a sufficient amount of Ca and increased amount of Mg, which allows the better development of individuals on serpentinite substrate; Ca and Mg have regulatory abilities for many enzymes, and influence the formation of structural changes in plants [81]. 

The results obtained in this study show that soil type and chemical composition of the substrate influence the variation of morphological characteristics of *Teucrium montaum* and contribute to a complete overview of the adaptive response to specific chemical properties of the substrate. Based on previous studies of *Teucrium* species [82], the geometric morphometrics approach provides new insights into their biology and ecology.

## 5. Conclusions

The results of the comparative analysis indicate that edaphic factors, especially metal content, influence the differentiation of the morphological traits of *Teucrium montanum*. The degree of phenotypic variation in the selected morphological traits differs between individuals sampled from serpentinite substrate, compared to individuals from calcareous substrate. By comparing the mean shapes of the examined plant structures, the differences between their shapes were determined, which were mainly manifested through the expansion and narrowing of certain structural elements. The main differences between the populations are the narrower part of the lower lip crown, the narrower leaf, and the wider central part of the vascular system in the serpentinite populations, unlike the calcareous populations. It has been shown that there are significant differences in size between leaves and stems from plant populations collected from calcareous and serpentinite substrates, as well as differences between the shape of the stem for the symmetric and asymmetric components concerning the type of substrate. The fluctuating asymmetry of the studied plant structures is potentially an indicator of stress on serpentinite habitats due to the specific chemical composition, which as such is a consequence of developmental instability. The differences in symmetric and asymmetric components between *Teucrium montanum* populations from different soils suggest that both genetic and environmental factors shape the morphology of this species. The observed morphological difference could represent a potential adaptive response to the specificity of the geological substrate. 

## Figures and Tables

**Table 1:** The geographical data of the studied *T. montanum* populations in the territory of Serbia.

No.	Locality/Abbreviation	Altitude	Latitude (N)	Longitude (E)
1 *	Miroč Mt., Mali Štrbac/MS	576 m	44°38′3.065″	22°18′48.022″
2	Homolje Mts., Ježevac/JE	675 m	44°16′0.396″	21°31′1.672″
3	Trešnjica Canyon/KT	476 m	44°8′40.877″	19°31′52.859″
4	Veliki Krš Mt./VK	872 m	44°9′34.597″	22°5′48.285″
5	Lazar’s Canyon/KL	368 m	44°1′70.940″	21°57′27.009″
6	Tara Mt., Rastište/RA	477 m	43°56′36.132″	19°21′30.533″
7	Rzav Gorge, Kotroman/KO	517 m	43°46′23.747″	19°27′51.856″
8	Rtanj Mt.—near Šiljak/RT	1323 m	43°46′21.516″	21°53′39.029″
9	Jelašnička Gorge/JK	373 m	43°16′43.619″	22°4′6.975″
10	Vidlič Mt., Basarski kamen/BK	1014 m	43°10′32.524″	22°40′13.964″
11	Maljen Mt., Divčibare/DI	873 m	44°5′12.199″	20°0′39.43″
12	Brđanska gorge/BR	325 m	43°59′24.545″	20°25′14.454″
13	Orovica Mt.—near Kablar Mt./OR	538 m	43°54′51.852″	20°7′40.082″
14	Zlatibor Mt., Kremna/KR	815 m	43°50′7.305″	19°37′7.45″
15	Zlatibor Mt., Smiljanski zakosi/SZ	1044 m	43°40′15.066″	19°42′19.579″
16	Ibar Gorge, Maglič/MA	305 m	43°36′46.218″	20°33′5.414″
17	Goč Mt., Kamenica/KA	447 m	43°33′22.878″	20°42′20.631″
18	Raška, Trnava/TR	516 m	43°17′19.665″	20°35′53.466″
19	Giljeva Mt., Krajinoviće/KJ	1264 m	43°11′4.859″	19°51′43.409″
20	Novi Pazar, Negotinac/NE	1095 m	43°6′23.842″	20°39′2.104″

* Substrate type: calcareous substrate (1–10); serpentinite substrate (11–20).

## Data Availability

The data underlying this article will be shared upon reasonable request to the corresponding author.

## Supplementary Materials

The following supporting information can be downloaded at: https://www.mdpi.com/article/10.3390/plants12122381/s1, Table S1: Descriptive statistics for the examined plant structures of the *T. montanum*; Table S2: Analysis of variance (ANOVA) for the centroid size of corolla, leaf and stem of *T. montanum*. df—degree of freedom, MS—mean value of sum of squares, F—value of F test, P—level of statistical significance; Table S3: Differences in the mean values of the centroid size of the examined plant structures of *T. montanum* in relation to the type of substrate obtained by the Tuckey test. Statistically significant differences (P ≤ 0.05) are bolded; Table S4: Principal component analysis (PCA) for symmetric and asymmetric variance of corolla; Table S5: Principal component analysis (PCA) for symmetric and asymmetric leaf variance; Table S6: Principal component analysis (PCA) for symmetric and asymmetric stem variance; Table S7: Multivariate regression of shape variables on centroid size of examined plant structures of *T. montanum*. %—percentage of shape variation dependent on size variation, P—level of statistical significance; Table S8: Discriminant analysis. The values of the Procrustes distances between the mean shapes of the investigated plant structures in relation to the type of substrate with and without the allometric component are shown. Statistically significant differences are indicated in bold.
