# Supplementary material for: Geological Substrate Effects on Teucrium montanum L. (Lamiaceae) Morphological Traits: Geometric Morphometrics Approach
Source: Plants (Basel). 2023 Jun 19;12(12):2381. doi: 10.3390/plants12122381 (PMC10301171; doi:10.3390/plants12122381)
Supplement: Supplementary file 1 [file plants-12-02381-s001.zip › plants-2425135-supplementary.pdf]

plants MDPI

**Table S1.** Descriptive statistics for the examined plant structures of the *T. montanum*.

| CS | Sample size | Mean | Min | Max | SD | SE |
|---|---|---|---|---|---|---|
| Corolla | 800 | 359.458 | 249.903 | 463.804 | 39.690 | 1.403 |
| Leaf | 1200 | 339.533 | 174.143 | 618.234 | 83.315 | 2.405 |
| Stem | 1200 | 190.603 | 104.637 | 340.391 | 37.156 | 1.073 |

**Table S2.** Analysis of variance (ANOVA) for the centroid size of corolla, leaf and stem of *T. montanum*. df - degree of freedom, MS - mean value of sum of squares, F - value of F test, P - level of statistical significance.

| Variation source | df | MS | F | P |
|---|---|---|---|---|
| Corolla | | | | |
| Geological substrate | 1 | 0.020 | 2 | 0.170 |
| Error | 798 | 0.010 | | |
| Leaf | | | | |
| Geological substrate | 1 | 17.960 | 396.0 | **0.000** |
| Error | 1198 | 0.050 | | |
| Stem | | | | |
| Geological substrate | 1 | 5.100 | 155.6 | **0.000** |
| Error | 1198 | 0.030 | | |

**Table S3.** Differences in the mean values of the centroid size of the examined plant structures of *T. montanum* in relation to the type of substrate obtained by the Tuckey test. Statistically significant differences (P ≤ 0.05) are bolded.

| | df | MS | Calcareous | Serpentinite | P |
|---|---|---|---|---|---|
| Corolla | 798 | 0.013 | 5.884 | 5.873 | 0.169 |
| Leaf | 1198 | 0.045 | 5.908 | 5.662 | **0.000** |
| Stem | 1198 | 0.033 | 5.291 | 5.160 | **0.000** |

**Table S4.** Principal component analysis (PCA) for symmetric and asymmetric variance of corolla.

| | Symmetric component | | | Asymmetric component | | |
|---|---|---|---|---|---|---|
| | Eigenvector ($10^{-5}$) | % Variation | % Cumulative | Eigenvector ($10^{-5}$) | % Variation | % Cumulative |
| $PC_1$ | 164.058 | 25.283 | 25.283 | 72.566 | 31.600 | 31.600 |
| $PC_2$ | 125.839 | 19.393 | 44.676 | 57.005 | 24.823 | 56.423 |
| $PC_3$ | 85.848 | 13.230 | 57.906 | 18.281 | 7.961 | 64.384 |
| $PC_4$ | 74.605 | 11.497 | 69.403 | 14.308 | 6.231 | 70.614 |
| $PC_5$ | 39.971 | 6.160 | 75.563 | 10.056 | 4.379 | 74.994 |
| $PC_6$ | 32.148 | 4.954 | 80.518 | 8.852 | 3.855 | 78.848 |
| $PC_7$ | 27.859 | 4.293 | 84.811 | 8.127 | 3.539 | 82.387 |

| | | | | | | |
|---|---|---|---|---|---|---|
| $PC_8$ | 21.654 | 3.337 | 88.148 | 6.662 | 2.901 | 85.288 |
| $PC_9$ | 16.933 | 2.610 | 90.758 | 6.436 | 2.802 | 88.091 |
| $PC_{10}$ | 13.502 | 2.081 | 92.838 | 5.907 | 2.572 | 90.663 |
| $PC_{11}$ | 10.872 | 1.676 | 94.514 | 5.438 | 2.368 | 93.031 |
| $PC_{12}$ | 10.566 | 1.628 | 96.142 | 4.881 | 2.125 | 95.157 |
| $PC_{13}$ | 9.233 | 1.423 | 97.565 | 3.896 | 1.697 | 96.853 |
| $PC_{14}$ | 5.939 | 0.915 | 98.481 | 2.950 | 1.284 | 98.138 |
| $PC_{15}$ | 5.382 | 0.829 | 99.310 | 2.487 | 1.083 | 99.221 |
| $PC_{16}$ | 4.478 | 0.690 | 100.000 | 1.790 | 0.779 | 100.000 |
| Total variance ($10^{-5}$) | | 648.888 | | | 229.642 | |

**Table S5.** Principal component analysis (PCA) for symmetric and asymmetric leaf variance.

| | Symmetric component | | | Asymmetric component | | |
|---|---|---|---|---|---|---|
| | Eigenvector ($10^{-5}$) | % Variation | % Cumulative | Eigenvector ($10^{-5}$) | % Variation | % Cumulative |
| $PC_1$ | 204.603 | 65.520 | 65.520 | 148.909 | 85.863 | 85.863 |
| $PC_2$ | 41.236 | 13.205 | 78.726 | 13.829 | 7.974 | 93.836 |
| $PC_3$ | 30.125 | 9.647 | 88.372 | 4.788 | 2.761 | 96.598 |
| $PC_4$ | 20.102 | 6.437 | 94.810 | 3.051 | 1.759 | 98.357 |
| $PC_5$ | 10.254 | 3.284 | 98.093 | 1.681 | 0.969 | 99.326 |
| $PC_6$ | 5.954 | 1.907 | 100.000 | 1.168 | 0.674 | 100.000 |
| Total variance ($10^{-5}$) | | 312.274 | | | 173.427 | |

**Table S6.** Principal component analysis (PCA) for symmetric and asymmetric stem variance.

| | Symmetric component | | | Asymmetric component | | |
|---|---|---|---|---|---|---|
| | Eigenvector ($10^{-5}$) | % Variation | % Cumulative | Eigenvector ($10^{-5}$) | % Variation | % Cumulative |
| $PC_1$ | 225.606 | 42.780 | 42.780 | 90.623 | 40.116 | 40.116 |
| $PC_2$ | 138.066 | 26.180 | 68.960 | 54.048 | 23.925 | 64.042 |
| $PC_3$ | 58.459 | 11.085 | 80.045 | 24.903 | 11.024 | 75.065 |
| $PC_4$ | 29.249 | 5.546 | 85.592 | 13.325 | 5.898 | 80.964 |
| $PC_5$ | 27.056 | 5.130 | 90.722 | 11.895 | 5.266 | 86.229 |
| $PC_6$ | 16.673 | 3.162 | 93.884 | 10.485 | 4.641 | 90.871 |
| $PC_7$ | 10.422 | 1.976 | 95.860 | 7.339 | 3.249 | 94.119 |
| $PC_8$ | 10.127 | 1.920 | 97.780 | 4.992 | 2.210 | 96.329 |
| $PC_9$ | 7.676 | 1.455 | 99.235 | 4.543 | 2.011 | 98.340 |
| $PC_{10}$ | 4.032 | 0.765 | 100.000 | 3.750 | 1.660 | 100.000 |
| Total variance | | 527.366 | | | 225.903 | |

| | |
|---|---|
| $(10^{-5})$ | |

**Table S7.** Multivariate regression of shape variables on centroid size of examined plant structures of *T. montanum*. % - percentage of shape variation dependent on size variation, P - level of statistical significance.

| | | % | P |
|---|---|---|---|
| Corolla | Symmetric | 5.829 | **0.000** |
| | Asymmetric | 0.394 | **0.009** |
| Leaf | Symmetric | 0.382 | **0.010** |
| | Asymmetric | 0.075 | 0.349 |
| Stem | Symmetric | 6.136 | **0.000** |
| | Asymmetric | 0.153 | 0.111 |

**Table S8.** Discriminant analysis. The values of the Procrustes distances between the mean shapes of the investigated plant structures in relation to the type of substrate with and without the allometric component are shown. Statistically significant differences are indicated in bold.

| | | **Procrustes distance** | | **Procrustes distance** | **Significance** |
|---|---|---|---|---|---|
| | | With allometry | P | Without allometry | P |
| Corolla | Symmetric | 0.017 | **<.0001** | 0.018 | **<.0001** |
| | Asymmetric | 0.004 | 0.228 | 0.018 | **<.0001** |
| Leaf | Symmetric | 0.007 | **0.015** | 0.009 | **<.0001** |
| | Asymmetric | 0.001 | 0.739 | 0.002 | 0.563 |
| Stem | Symmetric | 0.007 | **0.033** | 0.016 | **<.0001** |
| | Asymmetric | 0.003 | 0.343 | 0.004 | 0.120 |
